# Recent Advances Clarifying the Structure and Function of Plant Apyrases (Nucleoside Triphosphate Diphosphohydrolases)

**DOI:** 10.3390/ijms22063283

**Published:** 2021-03-23

**Authors:** Greg Clark, Katherine A. Brown, Manas K. Tripathy, Stanley J. Roux

**Affiliations:** 1 Department of Molecular Biosciences, University of Texas, Austin, TX 78712, USA; gbclark@utexas.edu (G.C.); kate01@utexas.edu (K.A.B.); 2 Cavendish Laboratory, University of Cambridge, J J Thomson Avenue, Cambridge CB3 0HE, UK; 3 Institute of Life Sciences, Bhubaneswar 751023, Odisha, India; mktripathy@gmail.com

**Keywords:** *Arabidopsis*, apyrase crystal structure, calmodulin, pea, potato, reactive oxygen species

## Abstract

Studies implicating an important role for apyrase (NTPDase) enzymes in plant growth and development began appearing in the literature more than three decades ago. After early studies primarily in potato, *Arabidopsis* and legumes, especially important discoveries that advanced an understanding of the biochemistry, structure and function of these enzymes have been published in the last half-dozen years, revealing that they carry out key functions in diverse other plants. These recent discoveries about plant apyrases include, among others, novel findings on its crystal structures, its biochemistry, its roles in plant stress responses and its induction of major changes in gene expression when its expression is suppressed or enhanced. This review will describe and discuss these recent advances and the major questions about plant apyrases that remain unanswered.

## 1. Introduction

Apyrases are nucleoside triphosphate diphosphohydrolases (NTPDases) that catalytically remove the terminal phosphate from NTPs and NDPs, but not from NMPs. They are documented in the proteome of all eukaryotes assayed, and are characterized by at least four conserved domains that include the nucleotide binding site [1]. They function both intracellularly, where their best-known activity is as an NDPase controlling the ADP level in Golgi lumen needed for protein glycosylation [2], and extracellularly, where they play key roles in limiting the concentration of extracellular NTPs and NDPs, which control diverse cellular activities in animals and plants [3]. Plant apyrases (APYs) were originally valued primarily for their low *K*_M_ for NTP and NDP nucleotides, and consequent utility in removing unwanted ATP from in vitro experiments (e.g., Ho et al. [4]), but they are now recognized to play key roles in controlling plant growth and development [5,6].

Although APYs function in spore-bearing plants [7], they have been the most studied thus far in flowering plants, and mainly in potato, *Arabidopsis* and legumes. They were originally characterized in potato [8], where there are seven family members [9], and on which ongoing research continues [10,11]. There are seven different APYs in *Arabidopsis*, and a recent review covered much of their known functions reported through early 2015 [12]. In legumes the main APYs studied are those in peas [13,14] and soybeans [15,16]. A recent report on another APY family revealed that there are nine different family members in wheat, and it provided novel information on the identification, characterization and role in stress responses of these monocot APYs [17]. Apyrases have been studied in many more plants than in potato, *Arabidopsis*, legumes and wheat, and there have been significant advances in understanding their structures and functions in recent years. This review will focus on these recent advances, mainly covering the literature published since 2015 on the biochemistry, structure, ectopic expression and physiological functions of APYs in diverse plant species. New findings on the value of APY inhibitors for enhancing the potency of pesticides will also be discussed.

## 2. Biochemical Properties of APY

After the early studies on potato APYs, most of the subsequent biochemical studies on plant APYs have been done on those purified from legumes and *Arabidopsis*. The first legume APY purified to near homogeneity and biochemically characterized was PsNTP9, which was isolated from nuclei of etiolated pea plumules [14]. This report characterized the *K*_M_, *V*_max_, molecular mass, substrate specificity and inhibitor sensitivity of this NTPDase, all of which were comparable to these properties previously reported for mammalian NTPDases. However, a unique property of this plant APY, not previously reported for any APY in animals was that its activity was significantly stimulated by Ca^2+^-activated calmodulin (CaM). A similar CaM-stimulated enzyme activity had previously been reported for a crude NTPase preparation isolated from pea chromatin [18].

Subsequently, the *Arabidopsis* AtAPY1 was heterologously expressed in bacteria, purified and found to have biochemical properties similar to those of the pea PsNTP9, including its stimulation by CaM [19]. Up to this time, the pea and *Arabidopsis* APYs that were characterized favored ATP as their substrate, but more recently Massalski et al. [20] reported evidence that crude preparations of His-tagged versions of AtAPY1 purified from light-grown *Arabidopsis* seedlings had little or no ATPase activity, and strongly favored ADP as a substrate. This finding supported the hypothesis previously proposed based on localization studies that AtAPY1 functioned mainly in the Golgi [21,22], where its ADPase activity would help regulate protein glycosylation, just as it does in yeast [2]. In support of this hypothesis, Chiu et al. [23] found that AtAPY1 could function as an endo-apyrase by complementing a yeast double mutant (-ynd1-gda1) that had no apyrase activity, and that microsomal preparations from this double mutant that expressed AtAPY1 favored UDP and GDP substrates.

The results of Massalski et al. [20] and of Chiu et al. [23] seemed to contradict prior studies that had shown that the AtAPY1 expressed heterologously in bacteria favored ATP as its substrate [19], and that the expression of AtAPY1 regulated the [eATP] of *Arabidopsis* cells during pollen tube growth [24], stomatal opening and closing [25] and seedling development [26]. Since the His-tag used by Massalski et al. [20] can alter the activity of an enzyme [27,28], and because post-translational modification of APYs can alter their substrate specificity [29], it became important to assess the substrate specificity of native, untagged AtAPY1 purified to near homogeneity from *Arabidopsis* tissues. These studies were carried out using APY extracted from purified nuclei of etiolated 3-d-old seedlings of either wild-type or *apy2* knock out seedlings of *Arabidopsis* [30,31]. These experiments used polyclonal antibodies specific to a unique 20-mer peptide of AtAPY 1 to verify that the final 50 kDa protein purified (>90% pure by silver stain) was AtAPY1. Activity assays indicated that the purified APY had very high specific activity for ATP (>7000 μM Pi/min/mg), no AMPase activity and favored ATP over ADP as its substrate.

The contrasting results of these studies on the NTPDase activity of AtAPY1 can be reconciled in a number of ways. Perhaps in light-grown adult tissues and in transgenic yeast most of the AtAPY1 is expressed in Golgi, where it has only NDPase activity, whereas in etiolated seedlings at least some of it is expressed in nuclei, where it has strong ATPase activity. Perhaps tagging AtAPY1 with poly-His enhances its NDPase activity, just as it enhances the activity of decapping scavenger enzymes in *C. elegans* [28], but this enhancement is not seen in the tag-less version of AtAPY1. Further studies will be needed to resolve this issue. Meanwhile it would be premature to assume that AtAPY1 cannot function as an NTPase in *Arabidopsis*.

It is likely that APYs carry out their functions in assemblies with other proteins, and learning, which proteins interact with APY will be critical for a fuller understanding of how they influence cell growth. Two recent reports have provided initial information on potential binding partners of AtAPY1. One used an immunoprecipitation/mass spectrometry-based approach to identify proteins associated with ROP1, a major regulator of the growth of *Arabidopsis* pollen tubes [31,32]. This proteomics study identified AtAPY1 as a potential interacting protein of ROP1. If this interaction is confirmed by independent studies, it would be of particular interest because pollen tubes release AtAPY1 as they grow, and blocking the function of this APY by specific antibodies inhibits pollen tube elongation [24]. A second report, thus far presented only as a meeting abstract, used a yeast two-hybrid approach to identify PATL4 (Sec14p-like phosphatidylinositol transfer family protein) as a potential AtAPY1-interacting partner [33]. That these two proteins could functionally interact would be consistent with the observations that both are involved in auxin polar transport, both are expressed primarily in rapidly growing tissues, and both have similar phenotypes when their expression is suppressed [5,34]. Nonetheless, additional studies would be needed for this AtAPY1-PATL4 interaction to be confirmed.

Two other APYs that were purified and biochemically characterized recently are those of poplar [35] and wheat [17]. The purified poplar apyrase, PeAPY2, favored ATP as a substrate and had a *K*_M_ of 390 µM. Its fusion protein, PeAPY2-eYFP, localized predominantly on the plasma membrane of transformed onion skin and *Arabidopsis* mesophyll protoplasts, where it was postulated to help regulate the [eATP] [35]. Like other APYs, the purified PeAPY2 was insensitive to inhibitors of P-, V- and F-type ATPases, such as NaF, Na_3_VO_4_ and Na_2_Mo_4_. However, it was also insensitive to NGXT191, unlike the *Arabidopsis* and potato APYs [24,36], but similar to the soybean GS52 APY [16], revealing there are species differences in the sensitivities of APYs to certain inhibitors.

Among the nine APYs genetically identified in wheat, the one biochemically characterized was the TaAPY3-1 protein, which was purified from a preparation heterologously expressed in *Escherichia. coli* without its transmembrane domain [17]. It had high catalytic activity for the hydrolysis of ATP and ADP, but it degraded TTP and GTP poorly. Its pH optima were in a lower range (4.5–5.5) than reported for pea and *Arabidopsis* APYs, and its *K*_M_ (8.7 mM) was much higher than that reported for the pea (0.6 mM), *Arabidopsis* (30 µM), soybean GS52 (424 µM) and *Populus* (390 µM) APYs, making it unlikely to be a major regulator of the [eATP], which would not be expected to reach the mM range [37]. The *K*_M_ values of the main NTPDases that control the [eATP] in mammals are in the 50–200 µM range [38,39].

## 3. Structural Organization of Plant Apyrases

**Figure 1:**
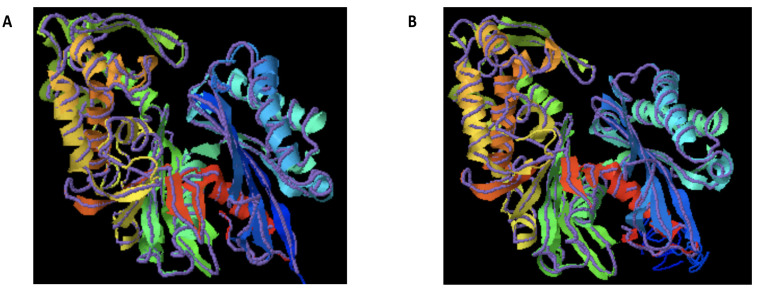
Models of selected plant APYs. Homology models of the potato (**A**) and pea PsNTP9 (**B**) apyrases are shown as ribbons colored with a rainbow spectrum. Both models are aligned with the crystal structure of the *V. unguiculata* APY crystal structure [41] show as a purple thread. Models and structural alignment were produced using the I-TASSER server [45,46,47].

Until recently knowledge about the three-dimensional organization of plant APYs was primarily based on homology models generated for potato APY [40], the soybean GS52 APY [16] and the *Arabidopsis* AtAPY1 [20]. These models were primarily based upon existing crystal structures of related enzymes from mammalian and bacterial species. However, in 2017 crystal structures were reported for two APYs from the plant species, *Vigna unguiculata* and *Trifolium repens* [41]. These crystal structures have revealed similarities in the fold and active site to structures obtained for the bacterial *Legionella pneumophila* NTPDase I [42] and mammalian NTPDase I and NTPDase II from rat [43,44]. Figure 1A shows a structural alignment between the *V. unguiculata* crystal structure and a new potato APY model (produced using the I-Tasser server; [45,46,47]). This alignment illustrates the presence of a two-domain structure, formed from two modified RNAase-H folding motifs assembled from α-helical and β-sheet subdomains [41]. This structural arrangement is also observed in the *T. repens* crystal structure [41] and the previously published plant APY homology models [16,20,40]. Figure 1B shows a similar structural alignment with a homology model for the pea PsNTP9 APY, retaining the same folding features.

**Figure 2:**
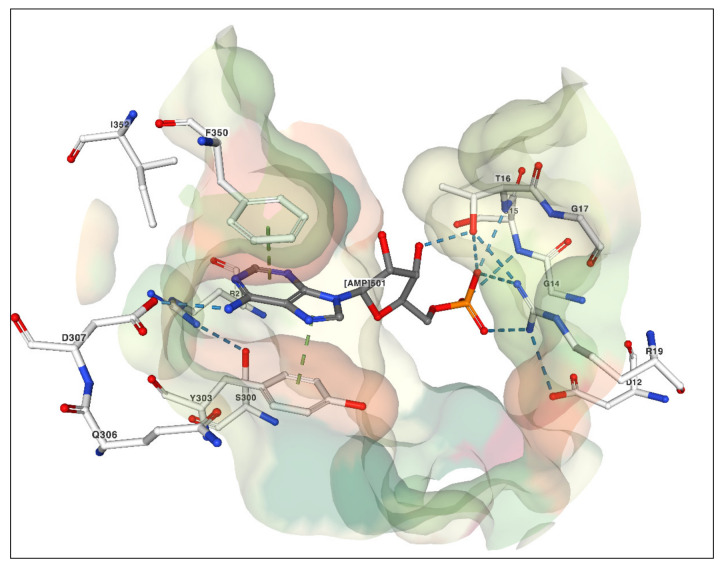
Active site of *T. repens* APY in complex with AMP. Residues in the active site and AMP (labeled [AMP]501) are shown in ball-and-stick representation (white bonds, residues; gray bonds, AMP). Interactions between residues and AMP are shown as green dashed lines within a rendered molecular surface. The adenine base of AMP is shown sandwiched between Y303 and F360 stabilized by π-stacking interactions. Residue D307 is also displayed. The area occupied by this residue in the active site is postulated to influence substrate affinities and specificities (see text and ref. [41]). This image was created using coordinates from RSCB PDB entry 5U7V [41] with the NGL viewer [52].

In addition to the conserved folds, all these enzymes contain the five apyrase conserved regions (ACRs), which are protein sequence motifs that are characteristic of NTDPases [48]. These ACRs contribute key structural features in the active site that play roles in substrate binding and turnover [49,50]. Within these ACRs, analysis of crystal structures of the *V. unguiculata* and *T. repens* APYs reveals that seven residues are likely to be critical for catalysis [41]. These residues are completely conserved in the sequences of the potato, soybean, pea PsNTP9 and AtAPY1 enzymes. The crystal structures also reveal that the base moieties of substrates bind to these enzymes using π-stacking interactions involving two aromatic residues (Figure 2) [41]. Pairs of the same aromatic residues are also present in the potato, soybean and pea PsNTP9 sequences. *Arabidopsis* AtAPY1 retains only one aromatic residue, a tyrosine, with the other position being an aspartic acid. This structural arrangement is similar to rat and *L. pneumophila* NTPDases, both of which use a tyrosine and a non-aromatic residue, arginine or asparagine, to “sandwich” nucleobases as observed in crystal structures of complexes with a variety of nonhydrolyzable NTP or NDP analogues [51].

Summers et al. [41] also presented comparative kinetic data for the *V. unguiculata* and *T. repens* APYs, demonstrating that these enzymes are capable of hydrolyzing NTP’s and NDP’s for all five bases (A, G, C, T and U) with varying apparent affinities, turnover number and pH optima. They suggested that differences in catalytic efficiency between these enzymes might be related in part to structural difference in the nucleotide binding pocket [41]. An aspartic acid residue present in *T. repens* structure (D307, Figure 2), and also present in the pea PsNTP9 sequence, was attributed to enable less selectivity between purine and pyrimidine bases. In the *V. unguiculata* structure, this residue is replaced by a glutamic acid residue, which is postulated to reduce affinity for larger purine substrates (NTPs) by increased steric bulk [41]. The AtAPY1, and soybean GS52 enzymes also have this conserved glutamic acid residue, while the potato APY has a glutamine in this position. Interestingly, characterization of the soybean GS52 APY showed a similar trend in its enzymatic activity, favoring smaller pyrimidines (NDPs) over larger purines (NTPs) [41]. It should be noted, however, that this enzyme is still able to turnover purine and pyrimidine NTPs and NDPs [16], as was observed for the *T. repens* and *V. unguiculata* enzymes [41]. It is therefore possible that the *Arabidopsis*, and potato APYs may exhibit a preference for smaller pyrimidine over larger purine substrates, although activity assays of these two APYs have standardly used ATP as the test substrate [9,19]. Taken together, these data suggest that all these plant enzymes possess conserved structural features that may allow for increased levels of base promiscuity than previously thought, and it would be premature to conclude that any of them are restricted to hydrolyzing only NTPs or NDPs. Additional studies are clearly needed to better establish structure–function relationships that influence their catalytic activities and substrate specificities.

**Figure 3:**
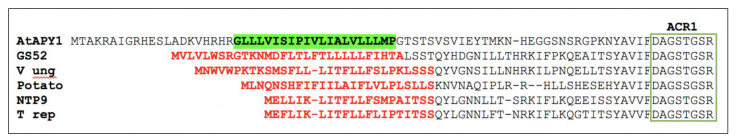
Sequence alignments of the N-terminal regions of plant APYs. The multisequence alignment was generated using Clustal Omega [55]. TOPCONS webserver [54] predictions are shown in bold. The predicted single-pass outward-facing transmembrane helix in the AtAPY1 sequence is highlighted in green. Bold red indicates predicted secretion peptide sequences. Relative positions of these N-terminal sequences to the ACR1 sequence motif (green box) are shown, the latter of which is present in the globular portion of all homology models and crystal structures.

Notably, these crystal structures and homology models lack structural information about the translated N-terminal regions. The original modeling studies of the potato APY identified this region to be a secretion sequence [40], while for the AtAPY1 model it was identified to contain a transmembrane helix [20]. Bioinformatic analysis of the cDNA clone encoding the soybean GS52 APY also predicted that N-terminal region contained a cleavable signal sequence [53]. These interpretations are consistent with the clearly extended length of the N-terminal region of the AtAPY1 sequence compared to that of the potato and soybean APYs (Figure 3). Furthermore, reanalysis of these regions using TOPCONS, a consensus prediction server for membrane protein topology and signal peptides [54], supports these earlier interpretations. As shown in Figure 3, the AtAPY1 sequence clearly suggests the presence of a transmembrane helix that would present the globular portion of this protein as an ectodomain. In comparison, the potato APY sequence shows primarily the presence of a signal peptide and weaker evidence of an inward-facing transmembrane helix, the latter of which could suggest a different trafficking pathway compared to the AtAPY1 enzyme. Analysis of the N-terminal sequences from the other plant APYs shows that they have the same pattern as that observed for the potato APY. These obvious differences may influence the localization of these enzymes, as ecto- or endo-APYs, and their cellular roles in the regulation of extracellular and/or intracellular levels of ATP/ADP.

## 4. Functional Studies on Plant APYs 

### 4.1. Studies Linking APY Expression to Growth Control

A role for eATP and APYs in regulating the growth of diverse plant cells and tissue types is well established [6]. Prior work has also revealed that eATP-mediated growth changes are dependent on downstream signaling steps, including changes in the levels of cytoplasmic Ca^2+^, NO and ROS, and they involve cross-talk with auxin and ethylene hormones [6]. Since cells release ATP as they grow, and high [eATP] inhibits cell growth, they need to limit their [eATP] to sustain growth, and ectoAPY is one of the major enzymes that serves this role [5,56]. For example, a recent transcriptomic study documented that the highest expression of an APY in *Brassica juncea*, BJAPY2, in expanding stems is under environmental conditions that most strongly promote stem growth [57]. Such studies have reconfirmed earlier reports that postulated a role for eATP and APYs in growth regulation using single cells such as pollen tubes [24,58], root hairs [59] and cotton fibers [60]. Likewise, a more recent study in *Nicotiana* confirmed the role of APY in pollen tube growth [61].

#### Correlating eATP Treatments and APY Effects on Growth

As noted in Section 3, the role of APY in regulating the [eATP] is not yet definitively resolved. However, recent studies have shown a correlation between eATP effects and altered APY expression on growth. These studies will be reviewed in this section.

Seedlings have been used often to illustrate how APY expression impacts the regulatory effects of eATP on plant cell growth. A recent study by Zhu et al. [62] linked eATP regulation of hypocotyl growth in light-grown *Arabidopsis* seedlings with the transcript abundance of the redox-responsive transcription factor 1 (RRTF1), whose expression is regulated by ethylene. In this study, treatment of light-grown *Arabidopsis* seedlings, which have a low level of APY expression in hypocotyls [24], with 500 μM ATP inhibited hypocotyl growth by 70%, but the same treatment of etiolated hypocotyls, which express a high level of APY, inhibits hypocotyl growth by only 20% [63]. These results reveal that endogenous APY levels alter the sensitivity of hypocotyls to the inhibitory growth effects of applied ATP.

Zhu et al. [62] also found that treatment of light-grown *Arabidopsis* seedlings with applied ATP inhibited the growth of primary roots, similar to the effects of suppressing APY expression, which raises the level of eATP [26]. The ATP treatment rapidly induced the expression of *RRTF1* in root tips, and was required for eATP-induced auxin accumulation in multiple root-tip zones, just as observed in APY-suppressed mutants [62,64]. Given the correlation of APY suppression and treatment with ATP with growth inhibition [26,63] and disrupted auxin transport [64], it would be of significant interest to examine whether the levels of *APY* expression and *RRTF1* expression are inversely correlated specifically in root tips.

Evidence that could favor a link between the expression of *APY* and *RRTF1* in controlling root growth would include the fact both APY suppression and increased RRTF1 expression inhibit root growth. However, these inhibitory growth effects may be mediated by different pathways. Recent qRT-PCR assays imply that enhanced RRTF1 expression would induce the expression of at least three ethylene-responsive transcription factors [65], which would be expected to inhibit root growth [66,67]. However, as judged by microarray analyses of whole seedlings (of which root tips would be a minor fraction), the suppression of APY expression did not significantly alter the transcript levels of either RRTF1 or the three ethylene-responsive transcription factors identified by qRT-PCR [65].

In his commentary on the Zhu et al. 2020 report on eATP induction of RRTF1, Chivasa [68], noted that recent experiments with the *dorn1* loss-of-function mutants indicate that there is another eATP receptor responsible for ATP-induced expression of *RRTF1* and inhibition of root growth in light-grown seedlings. This receptor could be the recently discovered P2K2 receptor [69] or an as yet undiscovered eATP receptor. That observation would be a good reason for future studies on the relationship between *RRTF1* and *APY* expression in root tips to include investigating how the expression of these genes is impacted by altered expression of the specific eATP receptors found in roots.

Both eATP treatment and altered APY expression impact the directional growth responses of primary roots, such as skewing and gravitropism. Yang et al. [70] found that overexpression of AtAPY1 and AtAPY2 promoted primary root growth, whereas disrupting their expression inhibited primary root growth. This study found that decreased expression of *AtAPY1*, chemical inhibition of APY activity or application of eATP increased root skewing. Correspondingly, seedlings overexpressing *AtAPY1* showed decreased skewing. Treatment of loss- and gain-of-function AtAPY1 mutants with NPA provided evidence that auxin transport plays a role in AtAPY1-mediated changes in root skewing. These results indicated a more prominent role for AtAPY1 than AtAPY2 in root skewing, and they are among the first to differentiate function between these two closely related *Arabidopsis* APYs.

Cells release ATP as they grow [24]. A recent finding, which was reported in a meeting abstract [71], more directly linked eATP levels to altered APY expression and growth. It used an ATP-specific microelectrode to record the [eATP] in the extracellular region within 2 µm of the root epidermis [72]. It showed that the primary roots of *Arabidopsis* seedlings have higher levels of eATP in their growth zones, where cells are releasing ATP as they expand, and that these levels are modified by altered expression of APY enzymes; i.e., the higher level of APY expression, the lower the [eATP].

The microelectrode studies would predict that APY expression in roots would impact Ca^2+^ signaling there, because in *Arabidopsis* the Ca^2+^ signatures induced by eATP treatment are dependent on the root cell type and region of the root [73]. This prediction was consistent with the data obtained in recent experiments that utilized a genetically encoded Ca^2+^ reporter, GCaMP3, under the control of different root cell-specific promoters to achieve the targeted expression of the reporter to columella, endodermal, epidermal, cortex or trichoblasts. The results indicated that the eATP-induced increase of [Ca^2+^]_cyt_ in root epidermal cells is more pronounced in the meristem region, where the expression of *AtAPY1* is higher than that of *AtAPY2*, than in the elongation zone, where the expression of *AtAPY2* is higher [24]. AtAPY1, but not AtAPY2, is calmodulin regulated [19], and so could be more likely to play a role in Ca^2+^ signaling than AtAPY2.

In addition to its regulation of root Ca^2+^ channels, eATP also appears to function in multiple conductance pathways in epidermal cells in the elongation zone of *Arabidopsis* roots [74], where APY expression is especially strong. Further insight on how eATP induces Ca^2+^ signatures in *Arabidopsis* roots was provided by a recent study that found AnnAt1 was a critical component of the enhanced Ca^2+^ uptake induced by eATP [75]. A valuable follow-up to these studies would be to investigate whether altered APY expression (constitutive or suppressed) in roots influences Ca^2+^-dependent signaling pathways in these organs.

One of the Ca^2+-^dependent signaling pathways in roots that could be regulated by eATP and ectoAPY was recently found to be modulated by phosphate nutrition. Matthus et al. [76] demonstrated that the eATP-induced increase in [Ca^2+^]_cyt_ in *Arabidopsis* primary roots was unchanged by phosphate starvation conditions at the root apex, where *AtAPY1* and *AtAPY2* are strongly expressed, but these conditions greatly reduced the eATP effect in the distal part of the root tip (≥1 mm from root apex), where much less APY is found. The authors suggested that this dampened calcium response to eATP in the distal part of the root may be due to the higher ROS and iron levels, which are induced to accumulate in this region of the root by phosphate starvation.

A follow-up study provided further insight into the complexity of the regulation of eATP-induced root Ca^2+^ signatures by nutrient availability [77]. This study found that iron starvation resulted in a heightened first apical [Ca^2+^]_cyt_ peak and a dampened and delayed subapical [Ca^2+^]_cyt_ peak, which were also linked to altered ROS levels. The suppression of *AtAPY1* and *AtAPY2* expression also induces higher ROS levels in roots [26], and thus the levels of these two APYs would be expected to impact the eATP-mediated Ca^2+^ signatures in *Arabidopsis* primary roots.

One way in which ectoAPY expression in roots could act jointly with eATP to enhance phosphate nutrition would be by hydrolyzing it and other extracellular nucleotides. Scheerer et al. [78] recently reported this potential role for ectoAPYs in beech and poplar roots, where their hydrolysis of NTPs in soil could generate phosphorous and nitrogen nutrients.

In a related study, Matthus et al. [79] used the GCaMP3 reporter to determine that the first apical increase in [Ca^2+^]_cyt_ induced by eATP causes the subapical increase, which is indicative of an eATP-induced “calcium wave” in roots of *Arabidopsis* seedlings. Since *AtAPY1* and *AtAPY2* are both highly expressed in seedling root apices, it is likely that their activity also contributes to regulation of this root “calcium wave”.

### 4.2. APY Control of Stress Responses

Abiotic and biotic stresses are often accompanied by growth inhibition and these stresses also regulate expression of APYs. For example, a study by Liu et al. [17] characterized the APY gene family in wheat and found their expression was differentially regulated by both abiotic and biotic stresses. A 12-h exposure to 300 mM NaCl upregulated all members of the gene family in wheat leaves, and several of them were rapidly upregulated also in wheat roots by the same treatment [17]. These results may indicate that APYs play an important role in salt stress responses in wheat, though further studies would be needed to resolve if and how they help plants overcome this stress.

Veerappa et al. [80] provided a more direct example of how APY expression can help enhance the resistance of plants to drought stress These authors found that ectopic expression of a pea APY, PsNTP9 (also known as PsAPY1) in *Arabidopsis* and soybean increased primary root growth, improved root system architecture (RSA) and increased seed yield under both normal and drought conditions [80]. Their RNA-seq analyses revealed changes in gene expression in the transgenic soybean lines that could help promote the improved RSA observed. Previous studies had found this pea APY localized in the ECM, Golgi membranes and the nucleus, and the authors proposed a model showing how PsNTP9 could potentially function in different locales to cause the resultant phenotypes. This pea APY appears to promote growth in tissues other than roots. For example, it is also highly expressed in pea apical hook cells during the various stages of their growth and development, as shown by Sharma et al. [81], using an mRNA in-situ hybridization assay.

APYs also help enhance plants resistance to the abiotic stress of cold. An ectoAPY from *Populus euphratica*, PeApy2, helps to meditate the response of this cold-tolerant tree to cold stress [35]. It is mainly localized on the plasma membrane, where the authors hypothesized it could help regulate the [eATP]. Its ectopic expression in *Arabidopsis* confers cold tolerance. One way proposed for how it does this is via increasing the rate of endocytosis/exocytosis, allowing for cold-damaged membranes to be repaired better. In support of there being a connection between the ectoAPY activity of PeApy2 and its regulation of endocytosis/exocytosis, the authors documented that treatment with high eATP inhibited while low eATP promoted endocytosis and exocytosis. They proposed that this regulation of secretory activity by eATP and ectoAPY would be required for the repair of cold-damaged membranes. This study also found that cold-stressed seedlings ectopically expressing PeApy2 had longer roots, which could in part be due to increased secretory activity required for cell growth.

A potential role for APY in salt stress responses is suggested by the finding that this response in *Arabidopsis* seedlings is mediated by eATP [82]. This report showed that eATP acts together with ethylene to make downstream Ca^2+^ and H_2_O_2_ signaling more robust [82]. It found that treatment with 300 µM eATP to salt-treated seedlings partially restored primary root growth in wild-type seedlings but not in the ethylene-insensitive mutants, *etr1-1* and *ein3-1eil1-1*. Additionally, eATP treatment increased transcript levels of *AtEIN3*, *AtEIL1* and *AtETR1* in salt-stressed roots. Another recent study linking eATP and ethylene signaling reported that eATP treatment of an *ein2* mutant altered the expression of 237 genes (mostly upregulated) by eATP treatment, which suggested that EIN2-dependent ethylene signaling suppresses the eATP-regulated expression of these genes [83]. 

The ectopic expression of certain APYs in transgenic plants is able to provide tolerance not only to abiotic stresses such as drought, cold and salt, but it may also provide some tolerance to biotic stress. Many reports have documented that eATP can serve a crucial signaling role in plant defense, which is mediated by the Dorn1/P2K1 receptor [84]. Recent characterization of P2K1-mediated gene expression clearly indicates an interplay between eATP, jasmonate, salicylic acid and ethylene defense hormones [85].

Both biotic and abiotic stress responses often include increases in H_2_O_2_ levels as intermediate signaling steps, and a recent study documented that eATP and APY can affect the response of cells to H_2_O_2_ [86]. In tobacco cell suspension cultures, treatment with H_2_O_2_ decreased cell viability and reduced both intracellular and extracellular ATP levels. Treatment with APY also decreased cell viability in response to H_2_O_2_-induced oxidative stress, but application of eATP partially rescued these phenotypes [86]. 

Toyoda et al. [87] summarizes a number of studies that have characterized the role of a pea APY, PsAPY1, in defense against fungal pathogen attack and links APY to the control of H_2_O_2_ levels in plants. This review followed up on an earlier discovery that PsAPY1 functions in the ECM of pea leaves in a large complex with copper amine oxidase [88], which mediates the H_2_O_2_ oxidative burst required for successful fungal infection. Treatment of the leaves with a fungal suppressor inhibits production of H_2_O_2_, and this inhibition required the coordinated regulation of both APY and copper amine oxidase enzyme activities in the APY complex. These results indicated that the PsAPY1-copper amine oxidase interaction might be important for regulating H_2_O_2_ levels during fungal attack [88].

Since ROS levels are intricately linked with biotic stress responses, and APY can help control these levels, it should not be surprising that APYs can be applied externally to help control these responses. Wang et al. [89] recently demonstrated that pretreating wounded bean leaves with apyrase reduced local and systemic levels of H_2_O_2_.

Beyond biotic stress responses, ectoAPYs are also demonstrated players in beneficial interactions of plants with microbes and fungi. As reviewed by Tanaka et al. [90], these beneficial interactions include symbiotic signaling guiding the process of nodulation and mycorrhizal associations. This review proposed a testable model hypothesizing that Nod factor-induced release of ATP combined with an increase in APY activity maintain eATP levels low enough to avoid the defense responses of legumes, and generate breakdown products like ADP, which may stimulate nodulation. 

Some pathogenic fungi suppress APY activity as part of their attack strategy. During the biotrophic phase of infection of pea plants, *Mycosphaerella pinodes* spores secrete two supprescins, which directly bind to the ectoAPY, PsAPY1 and induce its monomerization, inhibiting its activity. This appears to benefit infection by fungal spores by increasing eATP levels, which results in the activation of JA signaling, thus antagonizing the salicylic-acid signaling needed to respond to this early stage of infection [87]. 

Some insects also use APYs to suppress plant responses to their attack. A recent review highlights the similarities between blood pathogens targeting of APYs in animal cells with targeting of APYs by insect herbivores [91]. An example of this targeting was described in elephant grass, when it was attacked by the spittle bug (*Mahanarva spectabilis*), which has APY in its saliva. Both ATPase and ADPase activity was found in saliva collected from female spittle bugs, while male saliva samples only showed activity toward ATP [92]. The phytohormone response of elephant grass leaves, as evaluated by LC/MS, differed in response to mechanical damage and spittle bug infestation, showing that spittle bug saliva inhibited accumulation of jasmonic acid and zeatin levels. A model was proposed by which the APY in the spittle bug saliva can regulate plant eATP levels thereby manipulating the phytohormone response to insect attack.

## 5. Potential Value of Apyrase Inhibitors

Given the diverse ways APY expression can impact plant growth and development, inhibitors that could selectively block APY activity would be valuable tools for probing if and how the enzyme regulates various cellular activities. Finding such inhibitors did not occur quickly. In fact, APYs were originally distinguished from other ATPases because they were relatively insensitive to agents like oligomycin, azide, vanadate and molybdate, which had long been used to block ATPases whose function depended on ATP hydrolysis, such as ion transporters. Generic CaM inhibitors such as trifluoperazine and chlorpromazine were found to partially inhibit ecto-ATPase activity [93], and were used by Long et al. [94] as “NTPDase-specific inhibitors” to inhibit fungal infection of rice, but the inhibitory effects of these chemicals were too broad to be considered selective APY inhibitors. 

An initial attempt to identify more specific inhibitors of plant APYs utilized a colorimetric assay to select from a library of small compounds chemicals that could strongly inhibit potato APY while having a far less suppressive effect on the activity of other ATPases such as alkaline and acid phosphatases and luciferases [95]. The value of these inhibitors was demonstrated by Windsor et al. [36], who showed that they could enhance the potency of herbicides.

More recently, Tripathy et al. [96] tested whether some of the APY inhibitors selected by Windsor et al. [36] could be used to enhance the potency of fungicides. A strong rationale for the potential benefit of this study was that fungi constitute the largest number of plant pathogens, causing a wide range of plant diseases, and in current agricultural practices, many chemical fungicides have been used in high doses and frequent intervals to prevent crop losses [97]. Increasingly, however, fungi began to develop resistance to the most used fungicides, and countering this resistance and increasing the potency of current fungicides has become a major goal of modern agricultural research. 

Consistent with previous reports showing that APY inhibitors enhanced the potency of herbicides [36], Tripathy et al. [96] showed that several of these highly specific inhibitors also enhanced the potency of three different fungicides, copper octanoate, myclobutanil and propiconazole to suppress the growth of five different pathogenic fungi. The pathogen most sensitive to the increased fungicidal potency resulting from the inhibitor additions was *Sclerotinia*. This report tested 60 different treatment combinations, and found that in 53 of these combinations the sensitivity of fungi to the fungicide treatments was increased. Previous studies predicted that the effectiveness of the APY inhibitors was due to their ability to block the action of ABC transporters [95], which can export the fungicides from fungi. In accord with this prediction, fungi treated with propiconazole + APY inhibitor retained more propiconazole in the three different fungi tested than fungi treated with the fungicide alone.

These results were consistent with the findings that non-specific ATPase inhibitors can suppress deleterious fungal growth on rice [94]. They indicate that selective APY inhibitors may become valuable aids in efforts to reduce the heavy losses inflicted by pathogenic fungi on major crops each year.

As indicated in the above section on APY role in plant defense responses, plants use eATP as a wound signal, and insects and fungi release APYs as a way of blocking this signal and decreasing plants’ ability to resist biotic stresses. It is not surprising, then, that plants can produce chemicals that can inhibit the APYs used by insect and fungal attackers to blunt plant defense responses. Quercetin, a potent antioxidant flavonoid, was reported by Chen et al. [14] as a strong APY inhibitor, and recently other flavonoids, the chalcones, were also found to be strong APY inhibitors [10]. Thus, one of the functions of the chalcone synthases that are upregulated by biotic stresses [98] could be to produce compounds that inhibit APYs released by fungal and insect attackers.

## 6. Conclusions and Perspectives

Some of the key recent advances in research on plant APYs highlighted in this review are summarized in Table 1. They provide some perspectives on what goals of future research would be especially relevant. Following the discovery of an eATP receptor in mammalian cells, interest in ecto-NTPDases greatly increased, because these enzymes had the lowest *K*_M_ for ATP, and were thus likely to play a major role in limiting the [eATP] [99]. Similarly, interest in plant ecto-NTPDases, referred to as ecto-APYs, significantly increased with the discovery of a plant eATP receptor [84]. Although physiological data in *Arabidopsis* indicated that AtAPY1 and AtAPY2 might be prime candidates for serving as the main enzymes limiting [eATP] in these plants [24,26], several subsequent publications called this conclusion into question [20,21,22,23]. More recent biochemical [31] and structural [41] data have resurrected the possibility that AtAPY1 can bind and hydrolyze ATP, and thus could be classified as an NTPDase, at least in specific cells under specific growth conditions. New work on APY functions in peas [80], wheat [17], *Brassica* [57] and poplar [35] all underscore the central role of APYs in enhancing plant resistance responses to abiotic and biotic stresses. In many cases this enhanced resistance is due to APY functions in the plant cell wall [88]. Although their importance in controlling the [eATP] in these plants remains to be rigorously demonstrated, it is now clear that APYs have cross-talk with auxin and ethylene hormones, and play key roles in regulating the growth and development of diverse plants.

Major questions remain unanswered about APYs in plants. Do Golgi-localized APYs help control protein glycosylation? Do changes in APY levels impact changes in gene expression indirectly by controlling [eATP] and downstream changes in [Ca^2+^]_cyt_, or more directly through their function in nuclei? What are the binding partners of APYs, and how critical are they for APY functions? Do the binding partners of APYs differ depending on cell and tissue type? What structural features such as active site configuration, membrane localization and oligomerization control activities toward NTP and NDP substrates? We can anticipate that experiments planned or currently in progress will provide answers to these questions that will further substantiate the importance of APYs in the life of plants.

## Figures and Tables

**Table 1:** Key recent advances in research on plant APYs (2015–2020).

Advances	References
New crystal structures, insights on NTP binding domain	[41]
New evidence that APYs can hydrolyze ATP in ECM and nuclei	[30,35,88,90]
Initial identification of potential binding partners for APYs	[32,33,88]
New evidence for APY protective role in abiotic stress responses	[17,35,80]
New evidence for APY protective role in defense against pathogens	[87,88,96]
